# Iatrogenic Arterial Perforation During Endovascular Interventions

**DOI:** 10.7759/cureus.10018

**Published:** 2020-08-25

**Authors:** Tony Rizk, Darren Patel, Nahu G Dimitri, Khaled Mansour, Vijay Ramakrishnan

**Affiliations:** 1 Interventional Radiology, Edward Via College of Osteopathic Medicine, Blacksburg, USA; 2 Orthopedics, Edward Via College of Osteopathic Medicine, Blacksburg, USA; 3 Medicine, University of Medicine & Health Sciences, Basseterre, KNA; 4 Interventional Cardiology, Clinch Valley Medical Center, Richlands, USA; 5 Interventional Radiology, Clinch Valley Medical Center, Richlands, USA

**Keywords:** arterial injury, subclavian artery perforation, interventional radiology guided embolization, interventional cardiology, carotid stent, thyrocervical trunk, common carotid artery, external iliac artery, superficial femoral artery, covered stent

## Abstract

The use of minimally invasive endovascular procedures has increased, and as such, the frequency of associated vascular complications has also increased. Regardless of the access site location, rarely, arterial perforation can occur, which can be fatal if not properly managed. Interventionalists should be aware of the risk factors for perforation, commonly perforated vessels, and how different sites of perforation are diagnosed and managed. Rapid recognition and endovascular management reduce the need for open surgical repair, and thus reduce the morbidity and mortality of these complications. This review outlines the presentation, diagnosis, and management of iatrogenic perforations of the subclavian artery, thyrocervical trunk (TT), common carotid artery, superficial femoral artery (SFA), and external iliac artery.

## Introduction and background

In recent years, percutaneous endovascular procedures for peripheral and coronary vascular disease have become the mainstay of practice, replacing open surgical therapy [1]. During these procedures, catheters and guidewires are utilized when navigating through blood vessels. Analogous vascular and interventional radiology procedures have demonstrated less than 1% overall major complication rate and a shorter hospital stay [2]. However, the increased frequency of catheter-based therapy has led to an increased incidence of associated vascular complications. Although less invasive, these techniques can be associated with vessel perforation which is exceedingly rare but deadly [3].

Risk factors for vessel perforation include all comorbidities that promote medial artery calcification, such as hypertension, diabetes, and chronic renal failure, which lead to increased arterial stiffness [4]. Vascular anatomic variants and tortuosity, especially when utilizing a straight tipped wire, can predispose to vascular perforation [5]. During wire navigation, it is important that a catheter is advanced through vessels over a wire. Advancing a catheter without wire can cause trauma to the vessel when making contact with the open tip, resulting in dissection or perforation [6]. Moreover, the use of large sheaths has been associated with an increase in iatrogenic vascular access complications [7]. Table 1 summarizes the modifiable and nonmodifiable risk factors for iatrogenic vascular complications [7].

**Table 1:** Modifiable and Nonmodifiable risk factors for iatrogenic vascular complications.

Modifiable	Nonmodifiable
Large sheath size	Vascular calcification
Redundant guidewire manipulation	Vascular tortuosity
High angioplasty pressures	High-grade stenosis
Procedure time	Older age
Anticoagulation and thrombolytics	Diabetes mellitus
Long-term steroid therapy	Chronic kidney disease

The two most common locations of arterial vascular access are at the radial artery and the femoral artery. In general, the radial approach is favored due to less access site bleeding, definitive hemostasis, and quicker patient mobilization [5]. Complications associated with arterial access include spasm, hematoma formation, pseudoaneurysms, dissection, arteriovenous fistula, and distal ischemia [6]. Unlike the transradial approach, the femoral approach can lead to the extension of bleeding into the retroperitoneal space, which can be life-threatening [5]. Although the risk of major complications is significantly reduced via the transradial approach, large vessel perforation has still been reported in the literature for both approaches. 

Great vessel perforation is especially life-threatening due to the inability to provide pressure hemostasis, leading to an uncontrollable bleeding and hemodynamic collapse [8]. In addition, great vessel perforation can lead to sudden vessel closure, distal ischemia, hemorrhage into surrounding tissue with hematoma formation, and even death [1]. 

Therefore, rapid recognition and management are key to preventing patient mortality. This review serves to promote awareness of the common central and peripheral iatrogenic arterial perforation events that have been reported in the literature. By increasing awareness, clinicians will be better able to quickly recognize, diagnose, and manage such complications.

## Review

Subclavian artery 

Subclavian artery perforation is one of the more frequent iatrogenic central artery perforation events reported in the literature. These events more commonly occur when utilizing transradial access [5]. Relative to the femoral artery, the radial artery has a smaller luminal caliber and is more prone to vascular tortuosity, requiring straight tipped wire navigation. Straight tipped wires and stiff wires should be handled with delicate care to avoid trauma to the vessel wall. Perforations proximal to the aortic arch and near the origin of the vertebral artery are among implicated locations [5, 9]. Perforation at the first segment of the subclavian artery, which is located anterior to the cervical pleura and communicates with the mediastinum proximally, can result in mediastinal and cervical hematoma formation [10-11]. These complications are extremely rare and lethal, occurring in 0.008% of cases [11].

**Figure 1:**
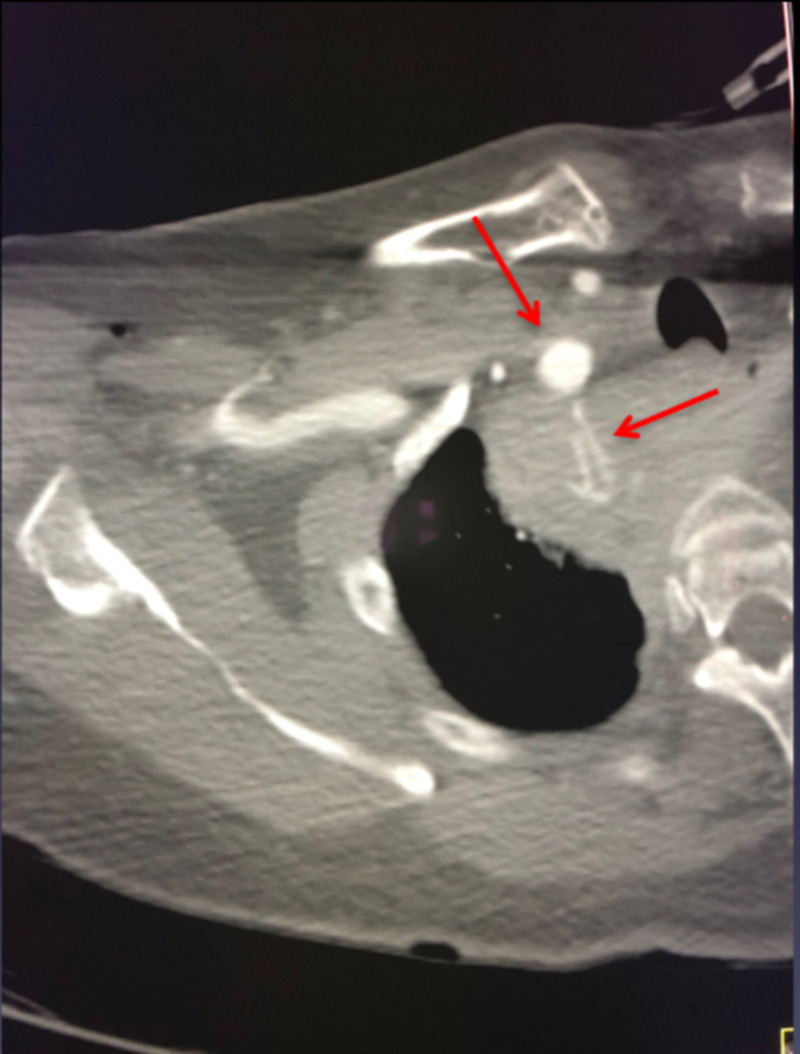
Axial CT angiogram of the chest showing active contrast extravasation from the right subclavian artery.

**Figure 2:**
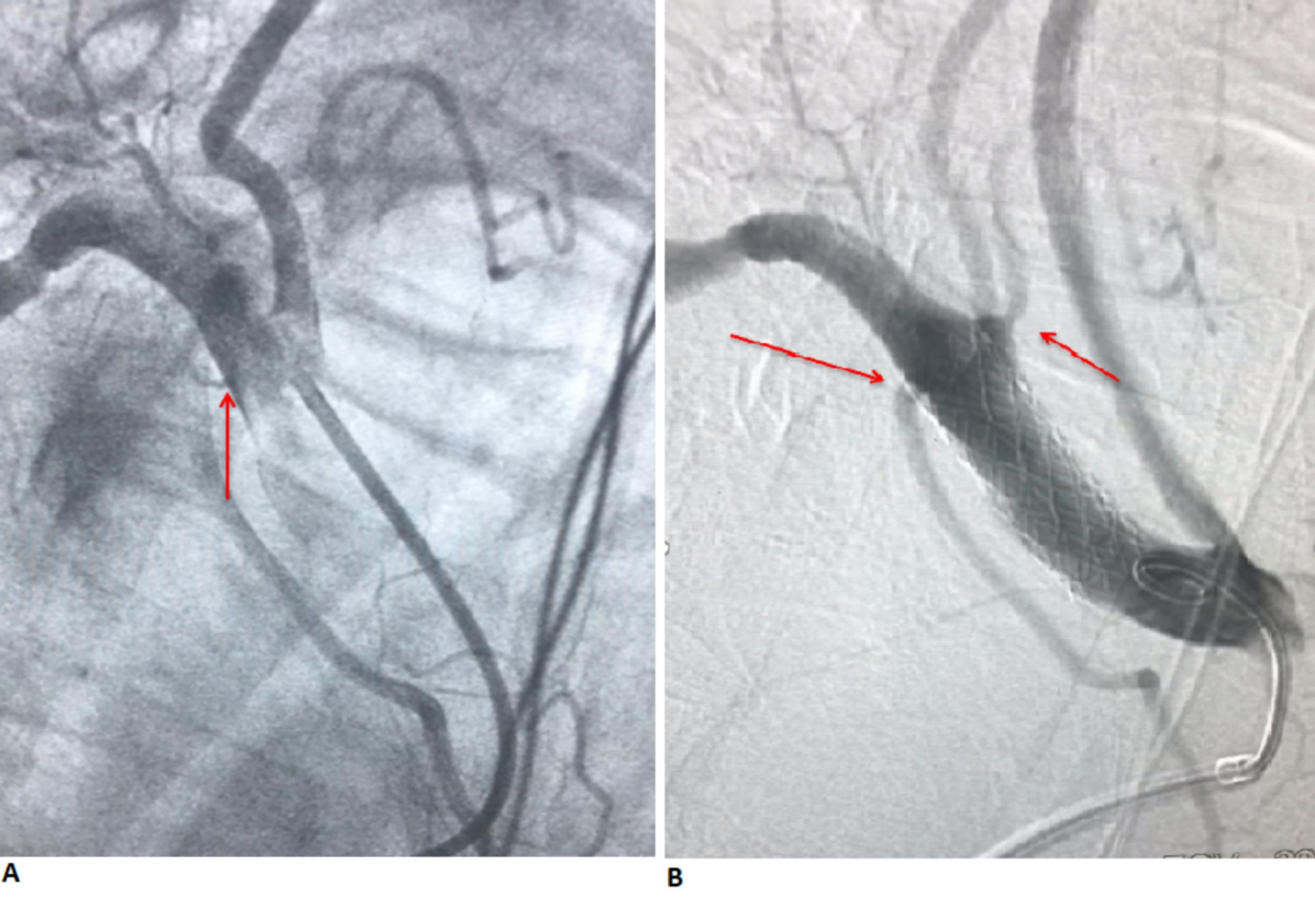
(A) Angiogram showing contrast extravasation from the right subclavian artery and the proximity of perforation to the origin of the right vertebral artery. (B) Angiogram showing stent placement with the cessation of contrast extravasation.

**Figure 3:**
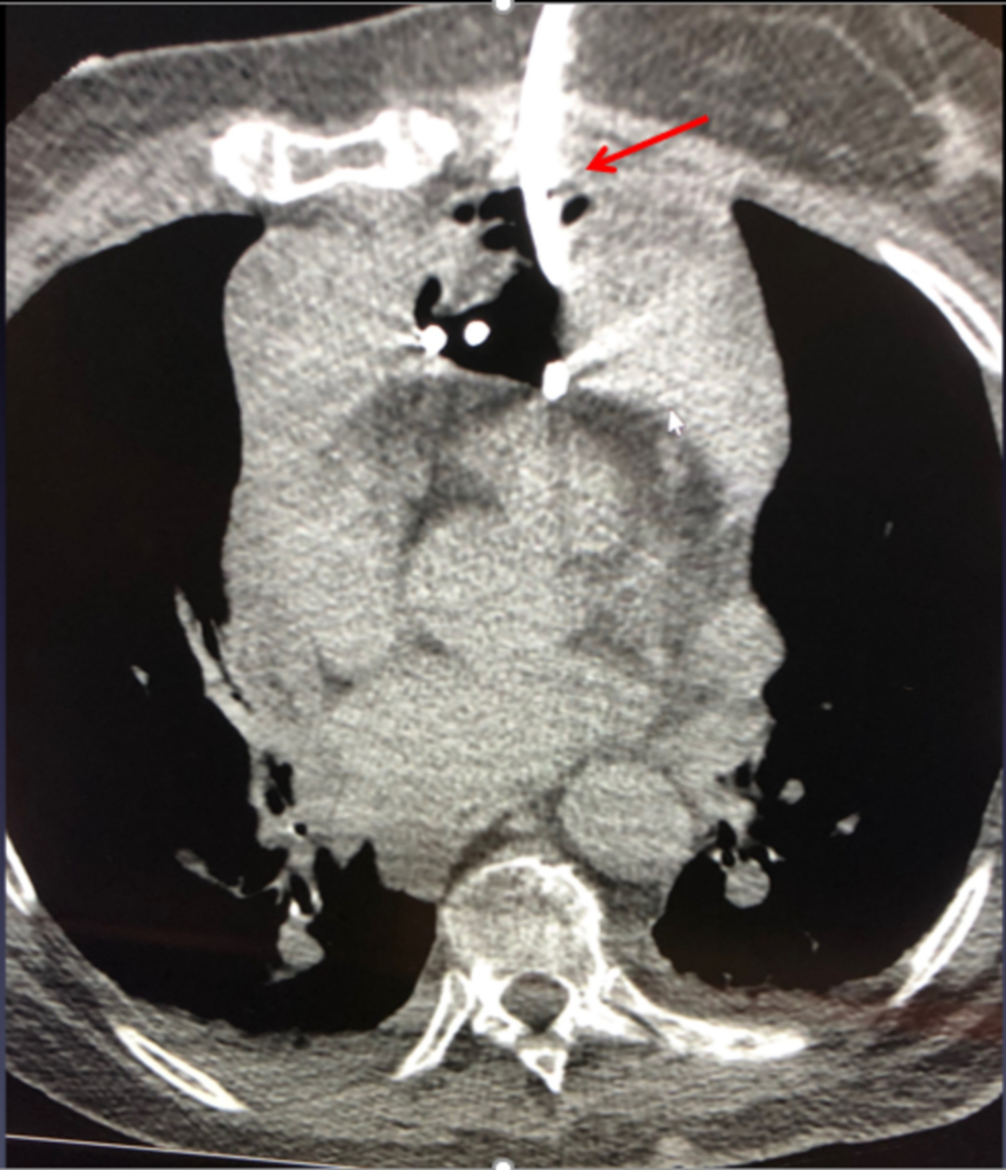
CT-guided drainage of mediastinal hematoma with an 8.5-French pigtail catheter.

Mediastinal hematoma presents as an obstructive shock following transradial access, as the blood accumulation mimics the mechanism of cardiac tamponade. When a patient presents with decompensation in this setting, rapid diagnostic imaging with a CT angiogram is imperative to locate the bleed in patients that can be resuscitated [5]. On CT angiogram, active contrast extravasation can be visualized from the subclavian artery (Figure 1), which requires rapid endovascular management. Endovascular management for subclavian artery perforation has demonstrated a 96.9% success rate, most likely due to the rapid and minimally invasive nature of the procedure [9]. Perforations of the subclavian artery can be managed with a balloon-inflatable covered stent, which allows both precise placement and occlusion of the perforation to restore adequate flow through the vessel (Figure 2) [12]. Successful endovascular management prevents the need for median sternotomy. Lastly, the resulting hematomas can be evacuated using CT guidance and pigtail catheters (Figure 3) [5].

Thyrocervical trunk

The thyrocervical trunk (TT) is a thoracic branch of the first segment of the subclavian artery. This trunk is located medial to the anterior scalene muscle, within the supraclavicular triangle, ascending cephalad and giving off four branches [13]. The four branches include the inferior thyroid artery, ascending cervical artery, suprascapular artery, and transverse cervical artery. The perpendicular and vertical position of this artery in relation to the subclavian artery can result in iatrogenic perforation during transradial percutaneous coronary intervention (PCI) [13].

Risk factors that may predispose to iatrogenic TT complications include radial artery spasm, atherosclerotic narrowing, and tortuosity of the innominate-subclavian artery junctions. These arterial features require hydrophilic wires during percutaneous transradial catheterizations [14]. However, due to the easy slippage of these wires, there is an inherently increased risk of vessel wall trauma to the branches of the right subclavian artery. In rare cases, slippage can lead to pseudoaneurysm and mediastinal hematoma formation due to the perforation of the TT in the first part of the right subclavian artery.

Perforation of the TT is one of the less frequent iatrogenic central artery perforation events reported in the literature, yet management is analogous to iatrogenic costocervical perforation during transradial percutaneous coronary intervention [15]. Case management begins with the identification of the TT perforation and accompanying pseudoaneurysm using subselection angiography of the TT [14]. Transcatheter coil embolization of the target vessel with micro coils and vascular plugs have been utilized to occlude the feeder vessel and stop the bleed [14]. This management has been shown to be well tolerated for TT wall trauma.

Carotid artery

Iatrogenic carotid artery injuries are frequently associated with the intended catheterization of the internal jugular vein [16]. Damage to the carotid artery can have serious neurological complications if not handled swiftly and effectively. Endovascular interventions, such as covered stent deployment, have become a better alternative to traditional surgical repairs of carotid artery perforation, rupture, and other neurovascular emergencies. Besides perforation with hemorrhage or rupture, other morphological injuries such as obstruction, pseudoaneurysm, fistula, and dissection of the carotid artery, do not require surgery [17]. Covered stent-grafts are commonly used for blowout syndrome, a complication of malignant processes in the head and neck that compromises and ruptures the carotid artery, in the setting of tumor invasion, but now feature in the repair of iatrogenic carotid injuries and in other emergency settings [16]. These repairs should be done with caution, as stents in the carotid artery come with the dreaded possible complication of in-stent restenosis [18]. Endovascular management of iatrogenic carotid injuries is preferred in contrast to the management of symptomatic carotid artery stenosis, in which carotid endarterectomy is the treatment of choice. 

Rupture, perforation, or dissection of the carotid injury may lead to rapid hemodynamic instability that requires an immediate CT angiogram of the neck to determine the presence of extravasation around the common carotid artery and any extensive hemomediastinum [17]. Using the femoral approach, angiography can then be utilized to locate the exact location of the extravasation, then a balloon-inflatable covered stent can be placed over the damaged segment [16].

Given the importance of the carotid artery to cerebral perfusion, neurological deficits are a feared complication, though DuBose et al. reports a success rate of 71.3% for endovascular stenting of the internal carotid artery with only 2.4% of new patients experiencing neurological dysfunction afterward [17].

Superficial femoral artery

Although the radial approach has taken the forefront for coronary artery angiography and intervention, femoral artery access is valuable for many procedures due to the larger size of the artery and corresponding acceptance of larger sheaths used in device placement for hemodynamic support [19]. Depending on the procedure and access availability, the superficial femoral artery (SFA) may be chosen in lieu of the common femoral artery (CFA) as a site of access, and with proper technique and management, remain complication-free. CFA cannulation is preferred over SFA due to the ability to provide pressure hemostasis over the puncture site using the underlying femoral head and less difficulty deploying closure devices. SFA access is preferred in patients with marked obesity, where CFA access would be difficult, and recent surgeries where CFA access is contraindicated [20]. Perforation of the SFA may occur due to improper angiographic technique or guidewire and angioplasty-related complications. 

When utilizing SFA access, proficient technique and high-quality angiography is required for road mapping and overlay to navigate the guidewire safely. By doing so, the operator will be aware when the guidewire enters a side branch, perforates the vessel wall, or inadvertently enters the vessel intima. Perforation of a vessel wall by guidewire can be small and generally do not result in significant bleeding. However, if a small side branch of the SFA is entered and perforated, it can lead to large thigh hematomas postoperatively [21]. Finally, angioplasty can also lead to perforation if it disrupts the adventitia of the vessel wall. Risk factors for angioplasty-related perforation include diabetes, older age, and the presence of critical limb ischemia. Additional factors also include heavy calcification of the vessel, high-pressure angioplasty, and oversized balloons [21]. Complications of SFA injury include compartment syndrome, pseudoaneurysm, and arteriovenous fistula formation.

Management starts with the identification of the perforation on an angiogram as extravasation, blush, or arteriovenous communication. The operator must discern the severity of the perforation, although no grading system exists for peripheral arterial perforation as it does for coronary artery perforation [22]. The first step in management includes balloon tamponade by placing a balloon across the defect and inflating to low pressure for two to four minutes. The balloon physically blocks bleeding and allows for initial coagulation to begin. If balloon tamponade is insufficient, a self-expanding covered stent may be considered due to the mechanical stress the SFA undergoes [21]. Finally, if a side branch vessel is perforated and is unable to be managed by the aforementioned techniques, coil embolization may be performed to control bleeding.

External iliac artery

Femoral access is favored for procedures requiring the use of larger sheaths such as transcatheter aortic valve implantation (TAVI), thoracic endovascular aortic repair (TEVAR), and endovascular abdominal aortic repair (EVAR) [23]. The use of larger sheaths carries an inherently increased risk of arterial injury. External iliac artery complications are potentially serious events that can present elusively due to its retroperitoneal location and may lead to hemorrhagic shock or death if they are not identified and managed rapidly. The reported incidence of retroperitoneal hematoma formation after interventional endovascular management is 0.49%-0.74% with a mortality risk of 4%-12% [7]. 

As with other peripheral arterial injuries, risk factors for external iliac artery injury include atherosclerotic disease, tortuous vessels, oversized balloons, history of recent endarterectomy, chronic steroid therapy, and diabetes mellitus [7]. Again, rapid identification on angiography with consideration of extravasation, surrounding structures, and retroperitoneal accumulation needs to be considered once an iliac artery perforation is identified. For external iliac artery perforation, balloon tamponade can serve as a temporary treatment for contained bleeding but is not definitive as the likelihood of rebleed remains due to vessel caliber and manipulation. Given the larger vessel caliber, endovascular management varies among the literature, but a general trend can be seen with the use of stent-grafts to manage iliac artery perforation [7]. 

Iatrogenic iliac artery injury may also occur during other vascular or orthopedic surgeries and successful management has been reported with endovascular repair. Adovasio et al. reported endovascular treatment of iliac artery injury following hip arthroplasty and hip prosthesis migration [24]. Canaud et al. also reported successful endovascular repair of an iliac artery injury following lumbar spine surgery [25].

## Conclusions

The increased use of arterial endovascular procedures in recent years has led to an increased frequency of their associated complications. In rare cases, wire navigation or catheter manipulation results in vessel wall trauma that may be of sufficient force to cause a deadly perforation. In general, once the perforated vessel is identified, immediate utilization of balloon tamponade along with the rapid reversal of anticoagulation and antiplatelet therapy is crucial. Simultaneously, any hemodynamic instability must be addressed to prevent further deterioration. As acute management is being administered, it is important to consult the appropriate surgical specialist if indicated. When feasible, the use of covered stents as treatment is preferred due to the significant morbidity of emergency vascular surgical repair. This review highlights different arteries implicated in iatrogenic injury, the importance of recognizing the risk factors for perforation, rapid recognition of these events, and appropriate diagnostic and interventional management that has been successfully implemented, with respect to vessel location corresponding anatomy. 
